# Surgical arthrolysis of the stiff elbow: a systematic review

**DOI:** 10.1007/s00402-022-04442-0

**Published:** 2022-04-28

**Authors:** Fabian Lanzerath, Kilian Wegmann, Michael Hackl, Stephan Uschok, Nadine Ott, Lars P. Müller, Tim Leschinger

**Affiliations:** Department of Orthopedic and Trauma Surgery, University Hospital Cologne, Kerpener Street 62, 50937 Cologne, Germany

**Keywords:** Elbow, Stiffness, Arthrolysis, Open, Arthroscopic, Surgical treatment, Outcome, Systematic review

## Abstract

**Introduction:**

Stiffness after elbow injuries can severely limit daily life. If adequate conservative treatment does not result in satisfactory improvement of elbow function, surgical intervention should be considered. Whether an open or arthroscopic procedure is preferable is still a topic of debate and a systematic review of functional outcomes is lacking.

**Materials and methods:**

We systematically reviewed the available literature searching electronic databases, MEDLINE using the PubMed interface and EMBASE, for studies published between 2013 and 2021. Primary objective was to compare open and arthroscopic arthrolysis’ functional outcomes, respectively, especially ROM and MEPS, as well as the accompanied complications. The PRISMA guidelines were applied.

**Results:**

27 studies comprising 1666 patients were included. 1059 patients (63.6%) were treated with open arthrolysis, and 607 patients (36.4%) were treated with arthroscopic arthrolysis. The results presented indicate satisfactory outcomes in open and arthroscopic arthrolysis with regard to functional outcome parameters. Treatment success, defined as excellent or good results according to the Mayo Elbow Performance Score, among the patients treated with an open procedure was 88.8%; 6.3% required revision whereas 18.1% had complications without the need for revision surgery. Within the cohort of arthroscopically treated patients, treatment success was 91.8%. Revisions and complications without further surgical intervention were significantly less frequent than in the open cohort, at 1.6% and 9.1%, respectively.

**Conclusions:**

Both open and arthroscopic arthrolysis provide good to excellent functional outcomes. Since the number of complications and revision increases with the invasiveness of the treatment, an arthroscopic procedure might be favored if feasible by indication. The role of forearm rotation and the use of a hinged external fixator remains of interest.

**Study design:**

Level IV; Systematic review.

## Introduction

The elbow joint is susceptible to stiffness due to the interlinking of three joints [1]. However, a functional range of elbow motion is essential to be able to perform most activities of daily living. Morrey et al. demonstrated that this is provided at a flexion–extension arc of 30°–130° and a forearm rotational arc of 50° supination and pronation, respectively [2]. Correspondingly, elbow joint stiffness is generally defined as a flexion–extension arc of less than 100° and/or flexion contracture of more than 30°. A movement restriction of 50° flexion–extension can result in a functional restriction of up to 80% of daily life [2]. The relevance of a functional range of motion becomes especially clear when one realizes that even the unrestricted use of a mobile phone requires an average flexion of 130° [3]. According to their etiology, elbow contractures can be classified as extrinsic, intrinsic, or a combination of both [4]. Extraarticular bone or soft tissue are typically responsible for extrinsic contractures, while intra-articular malunited fractures or osteophytes can cause intrinsic contractures [5]. Approximately, 12% of all traumatic injuries of the elbow cause stiffness and require surgical release [6]. Conservative treatment is primarily indicated for up to 6 months, including physiotherapy and dynamic or static progressive splinting. If adequate conservative treatment does not result in improved range of motion, if no functional range of motion is achieved, or patients have a higher functional demand than achieved, surgical intervention should be considered [7]. Depending on the status of the ulnar nerve, the formation and localization of heterotopic ossifications, the severity of contracture, the involvement of the articular surface, but also the individual skills of the surgeon, open arthrolysis or arthroscopic arthrolysis may be considered. The open procedure allows various approaches (lateral, medial, combined lateral and medial, posterior and anterior) and can optionally be supplemented by a hinged external fixator [8–11]. Especially during the last years, arthroscopic arthrolysis has gained popularity and was established a standard procedure.

A systematic review published in 2013 and performed by Kodde et al. presented the range of motion and complications in 698 patients treated openly and 100 patients treated arthroscopically [12]. However, functional outcome scores could not be assessed. We decided to perform an updated systematic review to gain a comparison of functional outcome scores, but also because since then, in particular, many arthroscopic treatment studies have been published.

## Materials and methods

The Preferred Reporting Items for Systematic Reviews and Meta-Analyses (PRISMA) guidelines were applied [13].

### Inclusion and exclusion criteria

Preliminary, the following criteria for inclusion were defined: Patients treated with either open or arthroscopic arthrolysis for elbow stiffness with a minimum of 1-year follow-up, clinical studies published in English or German language between 2013 and 2021, including at least five patients, providing preoperative and postoperative Range of Motion (ROM) and the Mayo Elbow Performance Score (MEPS). Articles whose functional outcomes directly related to fracture care, revisions arthrolysis, elbow ankylosis and arthroplasty were excluded. These criteria were also applied to studies included in the systematic review by Kodde et al., published in 2013 [12]. If the time period of the included patients of a study coincided with the time period of the included patients of another study published by the same first author, only one study was considered to eliminate redundant data.

### Search strategy

MEDLINE using the PubMed interface and EMBASE were searched for clinical studies using the MeSH terms elbow, stiffness and arthrolysis as key words. The search was performed on March 3, 2021.

### Study selection

**Fig. 1:**
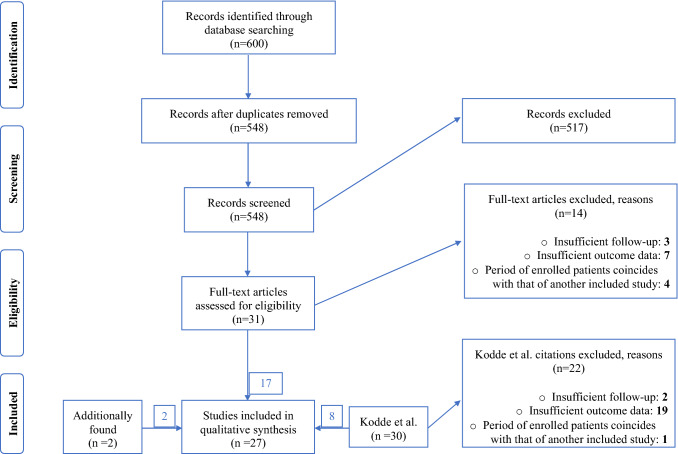
Study flow chart

The studies identified were independently scanned by two reviewers (F.L. and T.L.). At this stage, the titles and abstracts were assessed for eligibility. Full text records of the eligible studies were then analyzed. Full texts’ reference lists were additionally analyzed and searched for further articles, regardless of publication date. This procedure is illustrated in the PRISMA-adapted flow diagram (Fig. 1). Disagreement was resolved by consensus decision including a third reviewer (L.P.M.). Additionally, the studies included in the systematic review of Kodde et al. were evaluated [12]. However, only 8 of 30 studies originally included in the systematic review of Kodde et al. were eligible for inclusion. Reasons were insufficient follow-up in two cases, insufficient outcome data in 19 cases and the fact that the period of enrolled patients coincided with that of another included study published by the same research group in one case. By this means, a total of 27 studies comprising a total of 1666 patients were suitable for inclusion.

### Data extraction

Data of these 27 studies were extracted into prefabricated tables. Patients who underwent open surgery were compared with those who underwent arthroscopic surgery. Primary objective was to contrast functional outcomes between the open and arthroscopic approach, respectively, especially ROM and MEPS, as well as complication and revision rates. Secondary objective was to contrast functional outcome and complications within the open arthrolysis cohort depending on whether a hinged external fixator was used.

### Methodological quality

The methodological quality of each study included was assessed by assigning levels of evidence as previously defined by the Centre for Evidence-Based Medicine (http://www.cebm.net). Levels of evidence were assigned by 2 authors (F.L. and T.L.). If there was any disagreement, a third author was consulted (L.P.M.).

### Statistics

Treatment success, revision and complication rates were statistically compared between the open and the arthroscopic cohort using the chi-square test. The level of significance was defined as a *p* value of < 0.05 (SPSS; IBM, Armonk, NY, USA).

## Results

### Study selection

The initial search covered 600 publications published between 2013 and 2021. Removal of duplicates and exclusion of abstracts not fitting the inclusion criteria left 31 full texts for eligibility assessment. 14 were excluded. Reasons are outlined in the PRISMA-adapted flow diagram (Fig. 1). 17 publications were suitable for inclusion. The studies included in the systematic review by Kodde et al. were also supposed to be added; however, 22 of them were excluded because of reasons outlined in the PRISMA-adapted flow diagram [12]. Eight studies were suitable for inclusion. Additionally, two studies were found searching full texts’ reference lists. Overall, we included 27 studies. Among the included studies, four were retrospective cohort studies with a level of evidence of III. The level of evidence of the remaining 23 studies was Level IV.

### Study characteristics

Characteristics of the studies included are shown in Table 1. The study cohort of publications included was divided into two groups: Open arthrolysis and arthroscopic arthrolysis. Altogether, 1059 patients (63.6%) were treated with an open procedure while 607 patients (36.4%) were treated arthroscopically. Among the 1059 patients treated with open arthrolysis, the mean age was 37 years, mean follow-up was 37 months and 66% of the patients treated were male. Among the 607 patients treated arthroscopically, the mean age was 38 years, follow-up was 41 months, and the male proportion was 70%.

**Table 1:** Summary of the studies included

Author	Year	Technique	No. of pts	Mean age, years (range)	Mean FU, months (range)	Gender, % male
Cohen [9]	1998	O	22	35 (15–72)	26 (15–73)	55
Mansat [14]	1998	O	37	41 (5–68)	43 (24–74)	81
Ring [15]	2006	O	46	45 (18–79)	48 (24–108)	50
Sharma [16]	2007	O	25	34 (15–62)	94 (60–130)	76
Kulkarni [17]	2010	O	26	30 (12–60)	62 (42–113)	58
Park [18]	2010	O	42	37 (15–69)	39 (24–89)	48
Ayadi [19]	2011	O	22	31 (16–62)	56 (12–134)	80
Ouyang [20]	2013	O	11	42 (19–60)	29 (12–60)	64
Koh [21]	2013	O	24	39 (20–61)	60 (24–135)	46
Koh [22]	2013	O	77	38 (16–72)	42 (24–108)	56
Wang [23]	2015	O	18	34 (18–63)	20 (16–26)	56
Kruse [24]	2016	O	36	39 (19–58)	38 (24–58)	78
Chen [25]	2017	O	9	34 (13–52)	20 (18–22)	67
Zhou [26]	2017	O	38	37 (18–58)	31 (20–42)	55
Zheng [27]	2017	O	122	35	19 (12–29)	66
Gundes [28]	2017	O	77	35 (18–77)	44 (12–186)	69
Kwak [29]	2019	O	31	40 (31–49)	33 (27–33)	61
Sun [30]	2019	O	49	36 (18–58)	69 (62–83)	57
Sun [31]	2020	O	216	37 (18–65)	18 (12–25)	69
Xiong [32]	2020	O	131	35	47	81
**OPEN**			**1059**	**37**	**37**	**66**
Lapner [33]	2005	A	12	39 (28–50)	54 (12–120)	50
Nguyen [34]	2006	A	22	42 (13–67)	25 (12–47)	64
Kim [35]	2011	A	120	35	24 (24–24)	83
Pederzini [36]	2014	A	212	42 (9–65)	58 (36–84)	67
Wu [37]	2015	A	31	40 (19–51)	12 (12–12)	71
Lubiatowski [38]	2018	A	54	37 (13–68)	24 (24–24)	76
Rai [39]	2019	A	143	33 (12–58)	44 (24–90)	64
Kwak [29]	2019	A	13	39 (26–50)	27 (25–28)	85
**ARTHRO**			**607**	**38**	**41**	**70**

*ARTHRO* arthroscopy, *FU* follow-up, *O* open, *A* arthroscopic, *No.* number, *pts* patients

### Functional outcomes

A summary of functional results, including weighted means, is given in Table 2.

**Table 2:** Summary of the functional outcome

Author	No. of pts	Mean MEPS preop, points	Mean MEPS postop, points	MEPS detail	MEPS improvement, points	Flexionarc, preop, °	Flexionarc, postop, °	Gain flexionarc, °	Forearm rotation, preop, °	Forearm rotation, postop, °	Gain/loss of forearm rotation
Cohen	22	50	89	–	39	74	129	55	135	159	24
Mansat	37	62	81	15 E, 15 G, 1 F, 6 P	19	49	94	45	128	138	10
Ring	46	–	81	12 E, 19 G, 13 F, 2 P	–	45	103	58	94	141	47
Sharma	25	65	85	–	20	55	110	55	–	–	–
Kulkarni	26	45	89	13 E, 9 G, 3 F, 1 P	44	16	102	86	–	–	–
Park	42	73	94	32 E, 8 G, 2 F	21	55	115	60	–	–	–
Ayadi	22	–	76	6 E, 6 G, 6 F, 4 P	–	45	95	50	142	151	9
Ouyang	11	59	87	6 E, 3 G, 2 F	28	41	114	73	–	–	–
Koh	24	69	87	11 E, 10 G, 2 F, 1 P	18	60	105	45	–	–	–
Koh	77	–	92	–	–	45	112	67	–	–	–
Wang	18	62	97	15 E, 3 G	35	43	130	87	139	146	7
Kruse	36	44	91	–	47	52	109	57	–	–	–
Chen	9	58	94	6 E, 3 G	36	40	133	93	121	152	31
Zhou	38	68	96	30 E, 7 G, 1 F	28	27	126	99	148	154	6
Zheng	122	60	93	–	33	43	111	68	104	138	34
Gundes	77	60	85	–	25	45	110	65	170	180	10
Kwak	31	49	80	–	31	52	96	44	–	–	–
Sun	49	54	95	–	41	27	131	104	115	145	30
Sun	216	63	91	125 E, 80 G, 10 F, 1 P	28	40	118	78	76	128	52
Xiong	131	69	91	–	22	38	114	76	110	141	31
**OPEN**	**1059**	**61.4**	**89.7**	**434/489 (88.8%)**	**28.9**	**42.7**	**113.2**	**70.4**	**110.0**	**142.5**	**32.5**
Lapner	12	64	90	–	26	108	126	18	–	–	–
Nguyen	22	57	88	14 E, 5 G, 2 F, 1 P	31	84	122	38	150	148	–2
Kim	120	60	87	–	27	77	117	40	–	–	–
Pederzini	212	63	88	–	25	74	98	24	140	157	17
Wu	31	68	92	29 E–G, 2 F	24	49	115	66	–	–	–
Lubiatowski	54	73	93	–	20	–	–	33	–	–	–
Rai	143	63	90	73 E, 59 G, 10 F, 1 P	27	50	110	60	–	–	–
Kwak	13	52	81	–	29	72	107	35	–	–	–
**ARTHRO**	**607**	**63.1**	**88.8**	**180/196 (91.8%)**	**25.7**	**68.1**	**108.0**	**39.2**	**140.9**	**156.2**	**15.3**

*ARTHRO* arthroscopy, *No.* number, *pts* patients, *MEPS* Mayo Elbow Performance Score, *preop.* preoperative, *postop.* Postoperative, – not available, *E* excellent, *G* good, *F* fair, *P* poor

Mean MEPS improved from a preoperative 61.4 points to a postoperative 89.7 points in the open-treated cohort. The mean improvement was 28.9 points. Regarding patients who underwent arthroscopic surgery, the mean preoperative MEPS was 63.1 points, and the mean postoperative MEPS was 88.8 points, with an average improvement of 25.7 points. Excellent or good results concerning the MEPS were rated as successful treatment. Data regarding individual MEPS (excellent, good, fair or poor) were available in 489 patients treated by open surgery and 196 patients treated by arthroscopy. In 434 out of 489 (88.8%) patients treated with open surgery and in 180 out of 196 (91.8%) patients treated with arthroscopic surgery, the treatment was successful. Statistical analysis showed no significant difference between patients treated with open arthrolysis and patients treated arthroscopically concerning treatment success (*p* = 0.231). In the open cohort, the preoperative arc of motion improved from an average of 42.7° to an average of 113.2°, with an average improvement of 70.4°. In the arthroscopic cohort, the preoperative arc of motion improved from an average of 68.1° to an average of 108.0°, with an average improvement of 39.2°.

### Complications

Complications are summarized in Table 3. Complications were defined as ones reported as complications by the individual studies. Nerve symptoms were considered complications only if they were new-onset or exacerbated. If it was indicated to which nerve the respective complication referred, this is indicated in brackets in Table 3. If it was indicated whether the symptoms were transient or persistent, this is indicated accordingly in Table 3. Complications were not reported in all studies. The calculated average values were weighted according to study size.

**Table 3:** Summary of the complications

Author	No. of pts	No. of revisions	Ratio, %	No. of complications not requiring revision	Ratio, %
Cohen	22	0	0	4 transient nerve symptoms (3 ulnar, 1 median) 3 transient pain	31.8
Mansat	37	0	0	2 transient nerve symptoms (2 ulnar) 2 hematomas	10.8
Ring	46	7 arthrolysis 5 ulnar nerve neurolysis 2 arthrolysis and ulnar nerve neurolysis	30.4	–	–
Sharma	25	1 arthrolysis 1 ulnar nerve neurolysis	8.0	3 nerve symptoms (3 ulnar) 1 triceps avulsion	16.0
Kulkarni	26	1 ulnar nerve neurolysis and transposition 1 deep infection debridement 1 LUCL reconstruction (instability) 1 triceps lengthening 1 abdominal flap (wound dehiscence)	19.2	11 transient pin site infection 3 mild pain	53.8
Park	42	1 arthrolysis 1 deep infection debridement	4.8	1 contracture relapse	2.4
Ayadi	22	0	0	3 transient nerve symptoms (1 ulnar, 1 radial, 1 median) 1 persistent nerve symptoms (1 ulnar) 2 instability 7 MUA	59.1
Ouyang	11	0	0	2 transient nerve symptoms (1 ulnar, 1 radial) 1 transient pin site infection	27.3
Koh	24	1 arthrolysis 4 refracture	20.8	0	0
Koh	77	6 arthrolysis 1 superficial infection debridement	9.1	2 contracture relapse 1 instability	3.9
Wang	18	0	0	4 transient nerve symptoms (4 ulnar) 1 instability	27.8
Kruse	36	0	0	1 transient superficial infection	2.8
Chen	9	0	0	2 transient nerve symptoms (2 ulnar) 1 persistent nerve symptoms (1 medial antebrachial cutaneous)	33.4
Zhou	38	0	0	5 transient nerve symptoms (5 ulnar) 1 persistent nerve symptoms (1 ulnar) 2 transient pin site infection 4 pain 1 HO	34.2
Zheng	122	1 ulnar nerve neurolysis 3 infection debridement 1 LUCL reconstruction (instability)	4.1	10 nerve symptoms (8 ulnar, 1 radial, 1 median) 1 instability	9.0
Gundes	77	12 arthrolysis	15.6	10 transient nerve symptoms (10 ulnar) 4 superficial infection 1 HO	19.5
Kwak	31	1 arthrolysis	3.2	5 nerve symptoms (5 ulnar) 3 HO	25.8
Sun	49	0	0	4 nerve symptoms (4 ulnar) 3 transient pin site infection 2 HO	18.4
Sun	216	–	–	–	–
Xiong	131	0	0	20 nerve symptoms 9 instability 1 infection	22.9
**OPEN**	**1059**		**6.3%**		**18.1%**
Lapner	12	0	0	0	0
Nguyen	22	1 arthrolysis	4.5	1 transient nerve symptoms (1 ulnar) 3 portal tenderness	18.2
Kim	120	0	0	4 transient nerve symptoms (2 ulnar, 2 median) 1 reflex sympathetic dystrophy syndrome	4.2
Pederzini	212	2 ulnar nerve neurolysis and anterior transposition	0.9	2 transient nerve symptoms (2 radial) 1 persistent nerve symptoms (1 radial) 8 transient superficial infection 17 transient portal site synovial fistula	13.2
Wu	31	0	0	3 transient nerve symptoms (3 ulnar) 2 transient superficial infection 1 HO	19.4
Lubiatowski	54	1 arthrolysis 1 arthrolysis and ulnar nerve neurolysis 1 median nerve neurolysis	5.6	0	0
Rai	143	4 arthrolysis	2.8	4 transient nerve symptoms (4 ulnar) 3 transient superficial infection 1 contracture relapse 1 MUA	6.3
Kwak	13	0	0	2 nerve symptoms (ulnar) 1 HO	23.1
**ARTHRO**	**607**		**1.6%**		**9.1%**

*ARTHRO* arthroscopy, *No.* number, *pts* patients, – not available, *LUCL* lateral ulnar collateral ligament, *HO* heterotopic ossification, *MUA* manipulation under anesthesia

The revision rate of the open cohort was 6.3%. Common reasons were repeat arthrolysis and neurolysis with or without anterior transposition of the ulnar nerve. In 18.1% of the reported cases, complications without the need for revision surgery were reported. The revision rate of the arthroscopic cohort was 1.6%. Again, common reasons were repeat arthrolysis and neurolysis with or without anterior transposition of the ulnar nerve. 9.1% had complications not requiring revision surgery. The open cohort required significantly more revisions (*p* ≤ 0.001) and suffered significantly more complications (*p* < 0.001) than the arthroscopic cohort.

### Hinged external fixator

Table 4 compares open arthrolyses with respect to the use or nonuse of a hinged external fixator to improve stability. The study by Sun et al. (2020) was excluded from this analysis because it remains unclear which proportion, if any, was treated with a hinged external fixator.

**Table 4:** Comparison of open arthrolyses with regard to the use or non-use of a hinged external fixator

Author	No. of pts	Hinged external fixator	Mean FU, months (range)	Mean MEPS preop, points	Mean MEPS postop, points	MEPS detail	MEPS improvement, points	Flexionarc, preop, °	Flexionarc, postop, °	Gain flexionarc, °	Revision ratio, %	Complication ratio, %
Cohen	22	No	26 (15–73)	50	89	–	39	74	129	55	0	31.8
Mansat	37	No	43 (24–74)	62	81	15 E, 15 G, 1 F, 6 P	19	49	94	45	0	10.8
Ring	46	No	48 (24–108)	–	81	12 E, 19 G, 13 F, 2 P	–	45	103	58	30.4	–
Sharma	25	No	94 (60–130)	65	85	–	20	55	110	55	8.0	16.0
Park	42	No	39 (24–89)	73	94	32 E, 8 G, 2 F	21	55	115	60	4.8	2.4
Ayadi	22	No	56 (12–134)	–	76	6 E, 6 G, 6 F, 4 P	–	45	95	50	0	59.1
Koh	24	No	60 (24–135)	69	87	11 E, 10 G, 2 F, 1 P	18	60	105	45	20.8	0
Koh	77	No	42 (24–108)	–	92	–	–	45	112	67	9.1	3.9
Kruse	36	No	38 (24–58)	44	91	–	47	52	109	57	0	2.8
Gundes	77	No	44 (12–186)	60	85	–	25	45	110	65	15.6	19.5
Kwak	31	No	33 (27–33)	49	80	–	31	52	96	44	3.2	25.8
**Total**	**439**		**46**	**59.4**	**86.3**	**134/171 (78.4%)**	**27.1**	**50.2**	**107.6**	**57.4**	**9.8**	**14.2**
Kulkarni	26	Yes	62 (42–113)	45	89	13 E, 9 G, 3 F, 1 P	44	16	102	86	19.2	53.8
Ouyang	11	Yes	29 (12–60)	59	87	6 E, 3 G, 2 F	28	41	114	73	0	27.3
Wang	18	Yes	20 (16–26)	62	97	15 E, 3 G	35	43	130	87	0	27.8
Chen	9	Yes	20 (18–22)	58	94	6 E, 3 G	36	40	133	93	0	33.4
Zhou	38	Yes	31 (20–42)	68	96	30 E, 7 G, 1 F	28	27	126	99	0	34.2
Zheng	122	Yes	19 (12–29)	60	93	–	33	43	111	68	4.1	9.0
Sun	49	Yes	69 (62–83)	54	95	–	41	27	131	104	0	18.4
Xiong	131	Yes	47	69	91	–	22	38	114	76	0	22.9
**Total**	**404**		**38**	**62.0**	**92.7**	**95/102 (93.1%)**	**30.7**	**36.1**	**116.6**	**80.6**	**2.5**	**21.8**

*No.* number, *pts* patients, *MEPS* Mayo Elbow Performance Score, *preop.* preoperative, *postop.* Postoperative, – not available, *E* excellent, *G* good, *F* fair, *P* poor

439 patients were treated without a hinged external fixator and followed up for an average period of 46 months. The mean postoperative MEPS was 86.3 points. The revision rate was 9.8% and the complication rate of those who did not undergo revision was 14.2%. 404 patients were treated with a hinged external fixator and followed up for a mean period of 38 months. The mean postoperative MEPS was 92.7 points. The revision rate was 2.5% and the complication rate of those who were not revised was 21.8%.

## Discussion

Stiffness after elbow trauma is common at 12% and can severely limit daily life [2, 6]. Randomized controlled trials and prospective comparative studies with sufficient follow-up are still unavailable and thus evidence is limited to level III or IV studies.

Many authors postulate that arthrolysis, whether open or arthroscopic, may be indicated after failure of conservative therapy. However, until today, the optimal treatment remains debatable and no systematic comparison of the functional outcome between open and arthroscopic surgery has been performed.

Based on the synthesis of previously unavailable functional outcome scores, this systematic review supports the hypothesis that both open and arthroscopic treatment can provide equally satisfactory arthrolysis outcomes. The average follow-up period was more than 3 years for both techniques, indicating adequate short-term results. Regarding the functional outcome according to the MEPS, neither group showed statistically superior results (Table 2). Significant differences were found with respect to revision and complication rates (Table 3). The open cohort required significantly more revisions and suffered significantly more complications than the arthroscopic cohort. At this point, however, it must be critically noted that the indications for arthroscopic arthrolysis are narrower than those for open arthrolysis: Pronounced heterotopic ossifications with topographic proximity to vascular-nerve pathways, for example, preclude purely arthroscopic arthrolysis. In this respect, only selected patients are eligible for arthroscopic arthrolysis, which is likely to confound the comparability of results. In the future, it will be of interest to randomize patients with the same indication to one of the two procedures.

In 2013 Kodde et al. published a previous systematic review dealing with elbow arthrolysis [12]. They enrolled 798 patients in their study, of whom only 100 underwent arthroscopic treatment [12]. The functional outcome could not be recorded and compared systematically [12]. Based on our inclusion and exclusion criteria defined preliminary, we were able to include only 8 out of 30 studies previously analyzed by Kodde et al. In total, 27 studies comprising 1666 patients were included. Consistent with the study by Kodde et al., the preoperative ROM of the open cohort was smaller and the gain in ROM greater when compared with that of the arthroscopically treated cohort [12]. Within the open cohort, patients treated with hinged external fixation were stiffer preoperatively and the gain in range of motion was greater than of those treated without hinged external fixation (Table 4). The fact that preoperative ROM correlated negatively with the degree of invasiveness of the surgical treatment chosen, from arthroscopic to open to open with hinged external fixator, is consistent with the results of Kodde et al. and the opinion of many authors that the first treatment modality after failure of conservative treatment should be as minimally invasive as possible.

With regard to revisions and general complications, arthroscopic treatment might be the safest available procedure (Table 3). When comparing the techniques in regard to their revision rate, 6.3% of the open arthrolysis had to be revised, whereas 1.6% had to be revised in the arthroscopic group. This trend also applies to complications not requiring revision surgery: the latter are almost twice as frequent in the open cohort as in the arthroscopic cohort (18.1% vs 9.1%). With a complication rate of 5%, Kodde et al. similarly refer to arthroscopic arthrolysis as the safest surgical procedure [12]. However, Kodde et al. considered only 100 patients treated arthroscopically, thus our complication rates should be considered more robust.

The study by Kwak et al. was the only study included comparing arthroscopy with open arthrolysis for post-traumatic stiffness [29]. In terms of pain, ROM, and functional outcome, neither group proved superior [29].

Regarding the use of a hinged external fixator Kodde et al. reported a complication rate of 73% [12]. However, this statement is based on two articles comprising 49 patients and should be viewed with caution. The present review showed a revision rate of 2.5% and a complication rate not requiring revision of 21.8% among patients treated with hinged external fixation. These percentages contrast with those of open arthrolysis without a hinged external fixator: 9.8% revision rate and 14.2% complication rate not requiring revision. However, it should be noted that the follow-up period of the cohort with hinged external fixator was eight months shorter on average (38 vs. 46 months, Table 4). A complication rate of 73% as reported by Kodde et al. is therefore not to be concerned, nevertheless the standard use of a hinged external fixator should be further critically discussed. The implications of Table 4 require caution as all but one study that used a hinged external fixator in addition to the open procedure was performed at the same medical center where apparently a hinged external fixator is used as a standard procedure. [20, 23, 25–27, 30, 32]

The best approach for open arthrolysis is another point of interest for future research and could not be answered by the present work since individual data were lacking, making a recommendation impossible. Similarly, forearm rotation could not be analyzed systematically as it frequently lacked documentation.

The conclusion of Kodde et al. to treat as noninvasively as possible is supported by this systematic review, since the functional outcome is comparable, while the revision and general complication rates of the open procedure are higher. Nevertheless, the indications for an arthroscopic procedure remain limited, which becomes particularly evident when comparing the preoperative range of motion of the arthroscopic cohort with that of the open cohort (68.1° vs. 42.7°, Table 2).

Limitations of this systematic review include the retrospective design of all studies included and the lack of prospective randomized controlled studies comparing treatment options. In the future, it would be desirable to establish reporting guidelines to better evaluate the specific indication of the use or non-use of a hinged external fixator or to compare the effect of treatment on forearm rotation across studies.

## Conclusion

The functional results are satisfactory both after open and arthroscopic arthrolysis. The number of complications and revisions increases significantly with the invasiveness of the treatment. Among patients treated with open arthrolysis, 6.3% required revision surgery, whereas 18.1% suffered complications not requiring revision surgery. This contrasts with a revision rate of 1.6% and a complication rate without need for revision of 9.1% in the arthroscopically treated patient cohort. Therefore, within the feasibility of indication, an arthroscopic procedure might be favored. The indication scope of arthroscopic arthrolyses includes, for example, removal of osteophytes and capsulectomies. Extrinsic contractures and pronounced intra-articular ossifications, however, which may be difficult to access arthroscopically, are the domain of an open procedure. The role of forearm rotation and the use of a hinged external fixator remain pending.
